# Birth size and morphological femininity in adult women

**DOI:** 10.1186/s12862-020-01670-z

**Published:** 2020-08-15

**Authors:** Agnieszka Żelaźniewicz, Judyta Nowak, Bogusław Pawłowski

**Affiliations:** Department of Human Biology, University of Wrocław, ul. Kuźnicza 35, 50-138 Wrocław, Poland

**Keywords:** Weight, Ponderal index, Sexual dimorphism, Intra-uterine growth, Breast size, WHR

## Abstract

**Background:**

Women’s morphological femininity is perceived to develop under the influence of sex hormones and to serve as a cue of estradiol level, fertility and health in mating context. However, as the studies on direct relationship between femininity and sex steroid levels have reported mixed results, it is still not well understood what factors contribute to inter-women variation in morphological femininity. Epidemiological studies show that indicators of adverse conditions during intrauterine growth and development in utero, such as low birthweight or relative thinness at birth, influence women’s physiology ovarian functioning and may be associated with life-time exposure to estradiol in women. Thus, here we tested if birth parameters are also related with the level of morphological femininity in adult women.

**Results:**

One hundred sixty-five healthy women of mean age 28.47 years (SD = 2.39) participated in the study. Facial femininity was estimated based on facial width-to-height ratio (fWHR) and facial shape sexual dimorphism measured in the photos. Body femininity was estimated based on waist-to-hip ratio (WHR) and breast size. Birth weight and birth length were obtained from medical records and ponderal index at birth was calculated. No relationship between birth parameters and facial or body femininity in women of reproductive age was found, also when controlled for adult sex steroid levels and BMI.

**Conclusions:**

The results suggest that, although previous research showed that birth parameters predict reproductive development and adult oestradiol level, they do not explain the variance in morphological femininity in women of reproductive age, trait that is thought to be a cue of a woman’s estradiol level and fertility in mating context.

## Background

Sexual dimorphism, one of the most studied morphological traits as a putative cue to heritable fitness benefits [14, 17, 49, 65, 74], develops at puberty, under the influence of sex hormones. In women, estradiol promotes development of body and facial feminine traits, such as increased body fat around hips, buttocks, thighs, and breast, higher cheekbones, rounder forehead and eyes, smaller chin and nose [29, 45, 63]. As estradiol is a key fertility hormone [47], exerts immunostimulating [78], anti-inflammatory [58], cardio-protective [5], and anti-oxidant properties [71], morphological femininity is expected to signal a woman’s fertility and health. Furthermore, feminine traits are perceived as attractive [49, 55, 66], what supports their role as a biological cue.

However, although inter-individual differences in the level of morphological femininity have been argued to be linked with variation in reproductive hormone levels [55], research on the relationship between sex steroids and femininity has reported mixed results. Some studies have reported that estradiol level is positively correlated with body [34] and facial femininity [14, 45], whereas other studies have failed to confirm this relationship [62]. Also, in some studies, femininity has been shown to correlate positively with long-term health [21, 74], what might be mediated by health protective properties of estradiol, whereas in others no such relationship (both with actual health and health during puberty) has been found [65].

One explanation for these equivocal results may be that morphological femininity is rather related with life-time exposure to estradiol levels, but not necessarily with adult estradiol level, that may be altered in response to changes in BMI [51], diet [19, 37], level of physical activity and energy expenditure [30, 31]. As such, one may expect that morphological femininity level will be related with traits underlying life-time exposure to estradiol level. Human epidemiological studies, supported by animal models, show that conditions during intrauterine growth are one of the key factors shaping adult physiology [8, 11, 20, 44, 50]. Indices of foetal development, such as size at birth, have been shown to affect ovarian development [13, 27] and life-time ovarian functioning [32], as well as age at menarche and at menopause [1]. Furthermore, birth size has been also shown to be positively related with estradiol level in adulthood [18, 32, 33]. Birth weight was also found to be positively related with breast cancer risk in women ([2, 15, 26, 52]; however see for contradictory results: [70]). The suggested mechanism for this is life-time exposure to high levels of estradiol [60].

Thus, the aim of this study was to test if birth size contributes to the variation in women’s facial and body femininity in reproductive age. As intra-uterine development impacts adult physiology and may underlie life-time exposure to estradiol level, we hypothesized, that birth weight and adiposity (evaluated by ponderal index) are positively related with facial and body femininity in women of reproductive age. As adult body adiposity [22] and sex steroid levels [14, 34, 45] have been shown to contribute to the level of a woman’s morphological femininity, and also previous studies suggests that the level of morphological femininity may fluctuate following short time changes in estradiol level [45, 40, 54]; although see also [48] for negative results) we controlled for these variables in the analyses.

## Results

### Descriptive statistics

Mean values, standard deviations, and ranges of birth parameters, femininity measurements and sex hormones levels are presented in Table 1. fWHR correlated positively with facial shape sexual dimorphism (facial SD) (*r* = 0.38, *p* < 0.001; 95%CI [0.24;0.50]) and with body WHR (*r* = 0.27, *p* < 0.001; 95%CI [0.12;0.40]), but not with breast size (*r* = 0.02, *p* = 0.78; 95%CI [− 0.13;0.17]). Similarly, facial SD was positively related with body WHR (*r* = 0.22, *p* = 0.04; 95%CI [0.07;0.36]), but not with breast size (*r* = 0.06, *p* = 0.47; 95%CI [− 0.09;0.21]. Furthermore, body WHR was not related with breast size (*r* = 0.03, *p* = 0.70; 95%CI [− 0.12;0.18].

**Table 1:** Descriptive statistics (*N* = 165)

	M	SD	Min	Max
Age	28.47	2.39	25.00	34.00
Birth weight [g]	3377.53	449.74	2200.00	4820.00
Birth length [cm]	54.31	2.88	46.00	63.00
Ponderal index [kg/m^3^]	21.12	2.42	14.24	28.80
Pregnancy week at birth	39.30	1.04	37.00	42.00
fWHR	2.10	0.15	1.76	2.58
Facial SD	0.20	0.58	−1.97	1.81
WHR	0.73	0.05	0.63	0.98
Breast size	1.17	0.04	1.07	1.30
E2 [pg/ml]	34.68	17.53	5.00	110.00
tT [ng/ml]	0.52	0.20	0.28	1.85
BMI	22.30	4.04	16.34	41.85

### The relationship between birth parameters and femininity level

The results of zero-order correlations between birth parameters and femininity measurements are presented in Table 2. Additionally, we found no correlation between birth weight and tT (*r* = 0.13, *p* = 0.08; 95%CI [− 0.02;0.28]) or E2 (*r* = − 0.03, *p* = 0.69; 95%CI [− 0.18;0.12]). We also found no relationship between birth ponderal index and tT (*r* = 0.04, *p* = 0.57; 95%CI [− 0.11;0.19]) or E2 (*r* = 0.02, *p* = 0.82; 95%CI [− 0.13;0.17]). None of the facial or body femininity measurements was related with testosterone or estradiol levels (in each case *p* > 0.05). Furthermore, BMI was not related with birth weight (*r* = 0.07, *p* = 0.38; 95%CI [− 0.08;0.22]) or ponderal index (*r* = 0.07, *p* = 0.37; 95%CI [− 0.08;0.22]) but was positively related with fWHR (*r* = 0.31, *p* < 0.001; 95%CI [0.16;0.44]), facial SD (*r* = 0.27, *p* < 0.001; 95%CI [0.12;0.40]), WHR (*r* = 0.45, *p* < 0.001; 95%CI [0.32;0.56]), and breast size (*r* = 0.15, *p* = 0.049; 95%CI [− 0.003;0.29]).

**Table 2:** The results of zero-order correlations between birth parameters and femininity measurements (*N* = 165)

	LOG Birth weight			LOG Ponderal index		
	r	*P*	95%CI	r	*p*	95%CI
LOG fWHR	−0.12	0.12	[− 0.27;0.03]	− 0.10	0.20	[− 0.25;0.05]
LOG Facial SD	0.03	0.67	[−0.12;0.18]	− 0.07	0.38	[− 0.22;0.08]
LOG WHR	−0.02	0.83	[−0.17;0.13]	− 0.07	0.39	[− 0.22;0.08]
LOG Breast size	0.01	0.92	[−0.14;0.16]	0.05	0.53	[−0.10; 0.20]

The results of multiple regression analyses showed no relationship between birth weight and facial or body femininity, also when controlled for pregnancy week at birth, BMI and sex steroids levels (Table 3). The confirmed that fWHR (Table 3 – Model 1), facial SD (Table 3 - Model 2), and body WHR (Table 3 – Model 2) were related only with women’s BMI, but not with birth weight or sex steroids levels. Breast size was not related neither with birth weight, nor with BMI or sex steroid levels (Table 3 – Model 4).

**Table 3:** The results of regression analyses for the relationship between birth weight and the femininity measurements, controlled for pregnancy week at birth, BMI, and sex steroids levels. Bolded results are statistically significant (*N* = 166)

	*β*	SD (β)	t(159)	*p*
**Model 1** (dependent variable – fWHR)	F(5,159) = 4.64, *p* < 0.001, *adj. r*^2^ = 0.10			
Birth weight	−0.13	0.08	−1.56	0.12
Pregnancy week at birth	−0.07	0.08	−0.85	0.40
**BMI**	**0.33**	**0.07**	**4.37**	**< 0.001**
E2	0.02	0.07	0.28	0.76
tT	0.04	0.07	0.60	0.55
**Model 2** (dependent variable – facial SD)	F(5,159) = 2.68, *p* = 0.02, *adj. r*^2^ = 0.05			
Birth weight	0.03	0.08	0.38	0.70
Pregnancy week at birth	−0.04	0.08	−0.45	0.65
**BMI**	**0.28**	**0.08**	**3.58**	**< 0.001**
E2	0.01	0.08	0.19	0.85
tT	−0.02	0.08	− 0.27	0.79
**Model 3** (dependent variable – body WHR)	F(5,159) = 9.02, *p* < 0.001, *adj. r*^2^ = 0.20			
Birth weight	0.002	0.08	0.03	0.97
Pregnancy week at birth	−0.11	0.08	−1.50	0.14
**BMI**	**0.46**	**0.07**	**6.52**	**< 0.001**
E2	0.05	0.07	0.68	0.50
tT	−0.05	0.07	−0.70	0.48
**Model 4** (dependent variable – breast size)	F(5,159) = 1.79, *p* = 0.12, *adj. r*^2^ = 0.02			
Birth weight	−0.04	0.08	−0.53	0.60
Pregnancy week at birth	0.13	0.08	1.61	0.11
BMI	0.14	0.08	1.77	0.08
E2	−0.08	0.08	−1.05	0.30
tT	−0.08	0.08	− 1.00	0.32

Similar results were obtained for the relationship between ponderal index and measures of facial and body femininity, also when controlled for pregnancy week at birth, BMI and sex steroids levels (Table 4). The results showed that fWHR (Table 4 – Model 1), facial SD (Table 4 - Model 2) and body WHR (Table 4 – Model 2) were positively related with women’s BMI, but not with ponderal index at birth or sex steroids levels. Breast size was not related neither with birth ponderal index, nor with BMI or sex steroid levels (Table 4 – Model 4).

**Table 4:** The results of regression analyses for the relationship between birth ponderal index and femininity measurements, controlled for pregnancy week at birth, BMI, and sex steroids levels. Bolded results are statistically significant (*N* = 165)

	*β*	SD (β)	t(159)	*p*
**Model 1** (dependent variable – fWHR)	F(5,159) = 4.38, *p* < 0.001, *adj. r*^2^ = 0.09			
Ponderal index at birth	−0.11	0.07	−1.51	0.13
Pregnancy week at birth	−0.10	0.07	−1.36	0.17
**BMI**	**0.32**	**0.08**	**4.27**	**< 0.001**
E2	0.02	0.08	0.28	0.78
tT	0.03	0.07	0.43	0.66
**Model 2** (dependent variable – facial SD)	F(5,159) = 2.93, *p* = 0.01, *adj. r*^2^ = 0.05			
Ponderal index at birth	−0.09	0.08	−1.13	0.26
Pregnancy week at birth	−0.02	0.08	−0.22	0.83
**BMI**	**0.28**	**0.08**	**3.69**	**< 0.001**
E2	0.02	0.08	0.24	0.81
tT	−0.01	0.08	−0.17	0.86
**Model 3** (dependent variable – body WHR)	F(5,159) = 9.43, *p* < 0.001, *adj. r*^2^ = 0.20			
Ponderal index at birth	−0.09	0.07	−1.27	0.21
Pregnancy week at birth	−0.10	0.07	−1.48	0.14
**BMI**	**0.47**	**0.07**	**6.63**	**< 0.001**
E2	0.05	0.07	0.74	0.46
tT	−0.05	0.07	−0.66	0.51
**Model 4** (dependent variable – breast size)	F(5,159) = 1.77, *p* = 0.12, *adj. r*^2^ = 0.02			
Ponderal index at birth	0.03	0.08	0.42	0.67
Pregnancy week at birth	0.11	0.08	1.47	0.14
BMI	0.13	0.08	1.71	0.09
E2	−0.08	0.08	−1.07	0.28
tT	−0.08	0.08	−1.10	0.27

Comparison of women from the lowest and the highest tercile of birth weight showed no difference in fWHR (t (105) = 0.67, *p* = 0.51), face sexual dimorphism (t (105) = − 0.30, *p* = 0.76), body WHR (t (105) = 0.22, *p* = 0.82) or breast size (t (105) = 0.43, *p* = 0.66). Also, comparison of women from the lowest and the highest tercile of birth ponderal index showed no difference in fWHR (t (91) = − 0.69, *p* = 0.49), face sexual dimorphism (t (108) = − 0.66, *p* = 0.51), body WHR (t (108) = − 0.23, *p* = 0.82) or breast size (t (108) = − 0.84, *p* = 0.40).

## Conclusions

The results of our study showed that birth parameters were not related with morphological femininity in women of reproductive age. The results were similar when controlled for pregnancy week at birth, adult BMI and sex steroids levels. Thus, although previous research showed, that birth parameters predict women’s reproductive physiology [12, 13], ovarian development, and life-time exposure to estradiol level [18, 27, 28, 32, 33], the results of our study suggest that intrauterine development does not contribute to the variance in morphological femininity in women of reproductive age.

The results of our study are in line with the studies that failed to show the relationship between birth size and adult sex hormone levels [53, 57, 75]. Jasieńska et al. [32] showed a positive correlation between birth size and oestradiol level in women of reproductive age, based on repeated E2 measurements during menstrual cycle (in contrast to [75], who used only a single measurement of E2), but their study sample included individuals with very low birth weight (< 1500 g), what might result from genetic or developmental disorders that also impact postnatal development, and might have confounded the results. Furthermore, Jasieńska et al. [32] showed that the relationship between birth weight and E2 was in fact observed only in women with lower birth weight (< 3000 g), suggesting a non-linear relationship between birth size and ovarian functioning in adulthood. In our study we found no difference in terms of morphological femininity between women with birth weight or ponderal index from the lowest and the highest tercile.

Furthermore, the results of this study are in line with findings suggesting no or weak relationship between facial sexual dimorphism and sex hormone levels or health [6, 65], questioning popular and influential hypothesis that sexually dimorphic traits are valid cues of an individual’s biological condition [67]. Although facial femininity, breast size, and WHR, are expected to reflect sex steroids levels, and as such fertility in women [34, 35, 45, 59], research show that these associations are weak, and have been inconsistent across the few studies directly examining this relationship in women [4, 16, 24, 36, 42]. Here, we also found no relationship between morphological facial and body femininity and reproductive hormone levels, suggesting that femininity is not a cue of a woman’s fertility.

The only variable related with facial and body femininity in our study was BMI. Similar relationship was shown in a number of studies on facial sexual dimorphism, showing that BMI is positively associated with fWHR in adults [23, 43, 46]. Previous research have showed that men are perceived as more masculine if they appear taller and heavier, independent of how much their facial shape differs from women’s [25]. Although, so far, there have been no similar studies conducted for femininity in women, one may presume that morphological sexual dimorphism may develop in relation with height and adiposity. This may happen under the influence of somatotropic hormones like growth hormone [73] or factors secreted by adipose tissue, what might obscure the relationship between sexual dimorphism and birth parameters.

Lack of the relationship between birth parameters and morphological femininity in women of reproductive age may also result from the fact that not all children with intrauterine growth retardation (IUGR) are characterised by low birth weight (LBW). Some children are smaller at birth due to their genetic predispositions, not unfavourable intrauterine environment, and these children may not be at higher risk of developmental disturbances resulting in lower health in adulthood. Similarly, some children with IUGR may not be characterised with low birth weight, due to their genetic predisposition for greater height [64]. Furthermore, maternal undernutrition in late gestation may influence the offspring’s body proportion without affecting birth weight [3]. As such, birth weight or ponderal index may not be sufficient to identify children with IUGR [61]. Additionally, most children with IUGR in western societies undergo catch-up growth during infancy [10, 76], and children who experience accelerated growth in early postnatal life are in greater risk of developing health problems related with IUGR [7, 9, 56]. Research suggests that this rapid postnatal catch-up growth of LBW neonates is even more important for adult health than birth weight alone [39]. Thus, it may be possible that the relationship between birth parameters and morphological femininity in women of reproductive age may be detected only by comparing children with asymmetrical and symmetrical growth in utero, or children with LBW and catch-up growth with children without catch-up growth.

It is also possible that some other factors, not controlled in this study, influencing a woman’s physiology during development (especially during puberty), might impact the hypothesized relationship between birth parameters and morphological femininity. Such interaction among characteristics resulting from foetal programming (e.g. adult estradiol level) and from adult lifestyle, may be important in determining the relationship between size at birth and adult morphological femininity. For instance, in some countries, low birth weight is not related to an increased risk of atherosclerosis [69] or breast cancer [38], probably due to the lifestyle factors, such as high physical activity, diet and low body weight. A large, population-based study from Denmark has shown no effect of age at menarche on breast cancer risk after adjustment for childhood growth patterns [2]. Furthermore, childhood lifestyle has been shown to affect the prepubertal or pubertal sex hormones levels as physical activity is negatively related with estradiol levels in young girls [77]. On the other hand, Jasieńska et al. [33] showed that birth size was negatively related with a woman’s vulnerability to ovarian suppression in response to her life-style factors, thus possible differences in life-style among our participant during their ontogenetic development, should not obscure the hypothesized relationship between birth parameters and adult femininity.

The results of the study did not confirm our hypothesis on the positive correlation between birth parameters, indicating conditions during prenatal development that are related with life-time exposure to estradiol level, and facial and body femininity in women of reproductive age. As such, our study adds to a growing literature that questions current suggestions about the signal content of morphologically feminine traits.

## Methods

This research was a part of a broader project on women’s health, that included 209 participants (M_age_ = 28.51, SD = 2.37), recruited via social networks and information in the local media. Present study is one in a series, examining the relationship between women’s appearance, facial and body morphology and a wide range of measures of a woman’s biological condition. Data collected in the main project included information on hormone levels, oxidative stress and inflammation markers, blood biochemical markers, morphological measurements, and birth parameters. The protocol used to recruit participants and collect data was approved by the local ethical committee. For each participant, the general purpose of the study was explained and the written consent was obtained for participation in the study and use of data for scientific purposes.

### Study sample & procedure

Women were selected for participation if they met the following criteria: age between 25 and 34 years, regular menstrual cycles and no fertility problems (including polycystic ovary syndrome and endometriosis), nulliparous, no current or chronic diseases, no hormonal contraception and no medication for at least 6 months before the study. Furthermore, only women born at term (37–41), with no evidence for a syndromic, chromosomal, or infectious aetiology of low birthweight were included. Participants were invited to participate in the study between the second and the fourth day of menstrual cycle (early follicular phase).

Facial photographs were taken and body measurements were conducted. Women completed the study survey, containing questions on date of birth, past and current health problems, including hormonal disorders. Biochemical blood tests were also performed in order to verify general health status, and fasting blood sample was collected for hormonal analyses. Data on birth weight and length were obtained from the participants’ personal “health books”, containing records about birth size, health condition at birth, and any parturition problems. Ponderal index at birth was calculated as birth weight/(birth length)^3^ and expressed in kg/m^3^. Ponderal index may be a better indicator of a newborn nutritional status than birth weight, in the same way that BMI (rather than adult body weight) better reflects adult nutritional status.

Based on collected information and health measurements 44 participants were excluded from the analyses due to the following reasons: 1) other than early follicular phase of menstrual cycle (*N* = 5); 2) chronic health problems (*N* = 11); 3) being born as a twin (*N* = 2); 4) preterm births (*N* = 6); 5) incomplete data on birth parameters (*N* = 19); 6) testosterone level exceeding 3SD of the mean testosterone level (*N* = 1). The final sample included 165 women of mean age 28.47 years (SD = 2.39).

### Femininity measurements

Facial femininity was estimated based on facial width-to-height ratio (fWHR) and facial shape sexual dimorphism (facial SD) measured in the photos. Face images were taken under standardized photographic conditions with digital still camera (Nikon D7100 with Tamron SP AF 17-50 mm F/2.8 XR Di II LD IF camera lens). Camera-to-head distance and camera settings were held constant. Participants had no make-up, were asked to have a neutral facial expression, to remove glasses or earrings, and to wear a hairband.

fWHR was measured as the distance between the left and right boundary of the face (width) divided by the distance between the upper lip and the highest point of the eye-lid (height), following Stirrat & Perrett [72], using WebMorph. Lower fWHR values indicate more feminine face. Face-shape sexual dimorphism was measured from each photograph, using a vector analysis method [25], following methodology from Cai et al. [6], using code for R script by Holzleitner et al. (https://osf.io/98qf4/; R script for analysing sexual dimorphism scores following Scott et al. [68] and Komori et al. [41]). Lower score indicates more feminine face shape. An additional adult 50 male (M_age_ = 27.67 years, SD = 3.14 years) and 50 female (M_age_ = 25.92 years, SD = 1.85 years) faces (recruited from the same population) were used to build the model used to calculate sexual dimorphism scores.

Body femininity was estimated based on waist-to-hip ratio (WHR) and breast size. Circumferences of waist, hips, breast and under breast were measured to the nearest millimetre. WHR was assessed by the circumference of the waist divided by the circumference of the hips. WHR is inversely related with femininity. Breast size was assessed by the ratio of circumference of the breast to the under-breast circumference. Breast volume is positively related with femininity. Height and weight were measured and BMI was calculated. All measurements (waist, hips, breast and under breast circumference, weight and height) were taken twice and means of the two measurements were used in the analyses.

### Hormones levels analyses

In order to evaluate serum testosterone and estradiol levels fasting blood samples were collected into EDTA Vacutainers (BD®). Serum was separated by centrifugation then portioned into micro-tubes and stored at -80 °C until analyses.

Serum total testosterone (tT) concentration was assayed using ELISA commercial kits (Demeditec Diagnostics GmbH® cat. no. DE1559). Samples preparation and assay procedure were performed in accordance with the manual supplied with the kit. Samples, standards (included in each assay) were measured in duplicate. Optical density (absorbance) was measured using microplate reader (ASYS UVM, Biochrom®) set to 450 nm. Testosterone concentration was calculated from the standard curve and expressed in ng/ml. Inter- and intra-assay coefficient of variability (CV) were less than 10% and less than 4.2% respectively, indicating good test precision and repeatability.

Estradiol level (E2) was measured on Roche Cobas analyser in a certified analytical laboratory (DIAGNOSTYKA®), using Roche’s technology (ElectroChemiLuminescence methods). The serum estradiol concentration was expressed in pg/ml.

### Statistical methods

The values of ponderal index, fWHR, WHR, breast size, BMI, E2 and tT levels were log-transformed for normalisation purposes, due to their strongly skewed or leptokurtic distribution.

First, to explore the data we run zero-order correlation analyses between birth parameters and femininity measurements. Additionally, we ran zero-order correlation analyses between various measures of morphological femininity and between the measures of femininity and sex steroids levels. Subsequently, we verified if the relationship between birth parameters and femininity can be detected when controlled for the potential confounders that might impact femininity (body adiposity and adult sex hormones levels) or birth weight (pregnancy week at birth). Thus, we ran four multiple regression analyses with the measurements of femininity as the dependent variables and birth weight, pregnancy week at birth, E2, testosterone, and BMI as predictors. Another four regression analyses were run for the femininity measurements as dependent variables and birth ponderal index, pregnancy week at birth, E2, testosterone and BMI as predictors.

In order to verify possible non-linear relationship between birth parameters and adult morphological femininity, we have also divided participants into three groups, based on terciles of birth weight, and compared the values of femininity measurements between women from the first (< 3200 g) and the third tercile (> 3555 g), using t-test. We have also conducted similar analysis for birth ponderal index, comparing women from the first (< 19.97 kg/m^3^) and the third tercile (> 22.16 kg/m^3^) of birth ponderal index, in terms of morphological femininity, using t-test.

Analyses were performed with Statistica 12.0 software. The results were interpreted as statistically significant if *p* < 0.05.

## Data Availability

Data available at https://osf.io/625bh/?view_only=1779b50ee0fe41428dbd45b1e6ee5706

## Abbreviations

E_2_: estradiol level
tT: total testosterone level
fWHR: facial width-to-height ratio
WHR: waist-to-hip ratio
BMI: Body Mass Index
Facial SD: facial shape sexual dimorphism
SD: standard deviation
